# Hemicellulosa-derived *Arenga pinnata* bunches as free-standing carbon nanofiber membranes for electrode material supercapacitors

**DOI:** 10.1038/s41598-022-06619-4

**Published:** 2022-02-16

**Authors:** Rakhmawati Farma, Irma Apriyani, Awitdrus Awitdrus, Erman Taer, Apriwandi Apriwandi

**Affiliations:** Department of Physics, University of Riau, 28293 Simpang Baru, Riau Indonesia

**Keywords:** Energy science and technology, Materials science

## Abstract

Carbon nanofibers derived from lignocellulosic materials have become the most prevalent free-standing electrode material for supercapacitors due to their renewable and sustainable nature. This study used *Arenga pinnata* bunches (APB) as raw material for hemicellulose compounds to produce carbon electrodes through carbonization processes at 650 °C, 700 °C, 750 °C, and 800 °C, in the presence of flowing N_2_ gas. The variations in carbonization temperature resulted in carbon electrodes with surface morphology having a nanofiber structure with micro-meso pore distribution. According to the results, the carbonization temperature of 700 °C (APB-700) is the optimum temperature for producing electrode surface morphology with a combination of nanofiber, micro-and mesopore distributions, as well as specific surface area, specific capacitance, energy density, and power density of 1231.896 m^2^ g^−1^, 201.6 F g^−1^, 28.0 Wh kg^−1^, and 109.5 W kg^−1^, respectively, for the two electrode systems. This shows the combination of nanofibers and the distribution of micro-and mesopores produced with variations in carbonization temperature has the capacity to improve the performance of supercapacitor cells. Therefore, carbon nanofibers derived from *Arenga pinnata* bunches have the potential to be used as free-standing electrode materials for supercapacitors without employing doping, binder, electrospinning, and heteroatom template methods.

## Introduction

The energy crisis caused by repeated and increasing use of fossil fuels each year leads to global warming, greenhouse effects, and environmental damage^1–3^. Therefore, renewable energy resources and other eco-friendly energy storage technologies have been developed to reduce the occurrence of these harmful effects^4–6^. Supercapacitors are extremely promising next-generation energy storage devices and have received enormous attention globally due to their eco-friendly advantages, high power features, extended cycle capability and stability, simple working principles, and short charge time^7,8^. This device has been implemented in portable electronic devices, hybrid electric vehicles, memory backups, and industrial power systems^9^. The supercapacitors' performance and quality are influenced by the materials selected for the manufacture of electrodes^10^ and the main components of porous carbon material-based supercapacitors, including carbon nanotubes, aerogel carbon, carbon graphene, and carbon nanofibers^11^.

Carbon nanofibers are the most commonly used carbon-based electrode materials for supercapacitor energy storage due to their excellent features, including lightweight, high strength, good corrosion resistance, thermal conductivity, and high surface area^12^. Carbon nanofibers are derived from green resources (biomass) with high cellulose, lignin, and hemicellulose contents^13^ and are used to produce electrodes with a pore distribution of micropore and mesopore combinations, as well as high specific surface area and capacitance. The pore distribution and surface morphology of carbon electrodes influence the transfer of electrolyte ions. Macropores produced by carbon electrodes serve as channels for easy access to electrolytes; however, these pores are not primarily responsible for optimum specific capacitance. Meanwhile, mesopores serve as a diffusion medium for electrolyte ions, and micropores store electrolyte ions to increase the specific capacitance. Thus, an increase in capacitance is primarily due to an increase in the carbon electrode meso- and micropores^14^.

This study focused on the synthesis of carbon nanofibers as a free-standing electrode material for supercapacitors, by subjecting *hemicellulose-rich Arange Pinnata* bunches to carbonization at 650 °C, 700 °C, 750 °C, and 800 °C. Various carbonization temperatures have been investigated as a major aspect in converting waste biomass to carbon electrodes with nanofiber-rich morphological structure. The carbonization process aims to produce high-purity carbon electrodes, as well as to form pore wells and increase the diameter of the electrode’s internal pores. These objectives are achieved through the deosiation reaction occurring at the carbon electrode, which is responsible for the decomposition of compounds found in biomass as well as the formation of new compounds through the chemical activation process. This reaction occurs completely at a carbonization temperature of 600–700 °C^15^, and any carbonization temperature above 700 °C is bound to cause excessive deosiation and consequently, damage the carbon electrode. High-purity carbon electrodes with nanofiber structure and adequate pore wells tend to have high material conductivity and high specific surface area, thereby improving the performance of supercapacitor cells.

*Arange pinnata* is a palm species that originated from East India and is widely distributed across Southeast Asia, including Indonesia, Malaysia, Thailand, and Myanmar. The species is often cultivated by farmers but is also reported to have been growing wild in tropical Asia for hundreds of years, due to its high adaptability and survival rate^16^. *Arange Pinnata* is a brown sugar-producing palm in great demand by Indonesian people. Generally, almost all parts of *Arange Pinnata* trees including the roots, stems, leaves, and fruits, serve as a source of income, as well as a source of raw material for the production of nira, starch, or flour^17^. However, the fruit’s bunches which are often regarded as waste, have a high lignocellulose content comprising 61.76% cellulose, 71.78% hemicellulose, and 23.48% lignin^18,19^. In biomass, high lignocellulose content, specifically hemicellulose, indicates a high carbon content, and suitability for use as a raw material for the manufacture of carbon nanofibers as a free-standing electrode material without heteroatom doping, binder, electrospinning, template method composites, or other synthetic materials^20^. This shows carbon electrodes prepared are bound to have a nanofiber structure with a precise pore size distribution, a large surface area, and high specific capacitance.

## Materials and methods

### Preparation of carbon electrodes

This study used an experimental methodology where the biomass of *Arenga pinnata* bunches obtained from the Department of Physics, University of Riau, Indonesia, were used as raw material for the fabrication of carbon electrodes. *Arenga pinnata* is an easily cultivable and highly sustainable species because the plant thrives well in tropical regions. According to the ICUN (Red List of Threatened Species https://www.iucnredlist.org/), this species is not classified as rare or endangered. However, further cultivation is recommended to increase the availability of nira and fruit.

**Figure 1:**
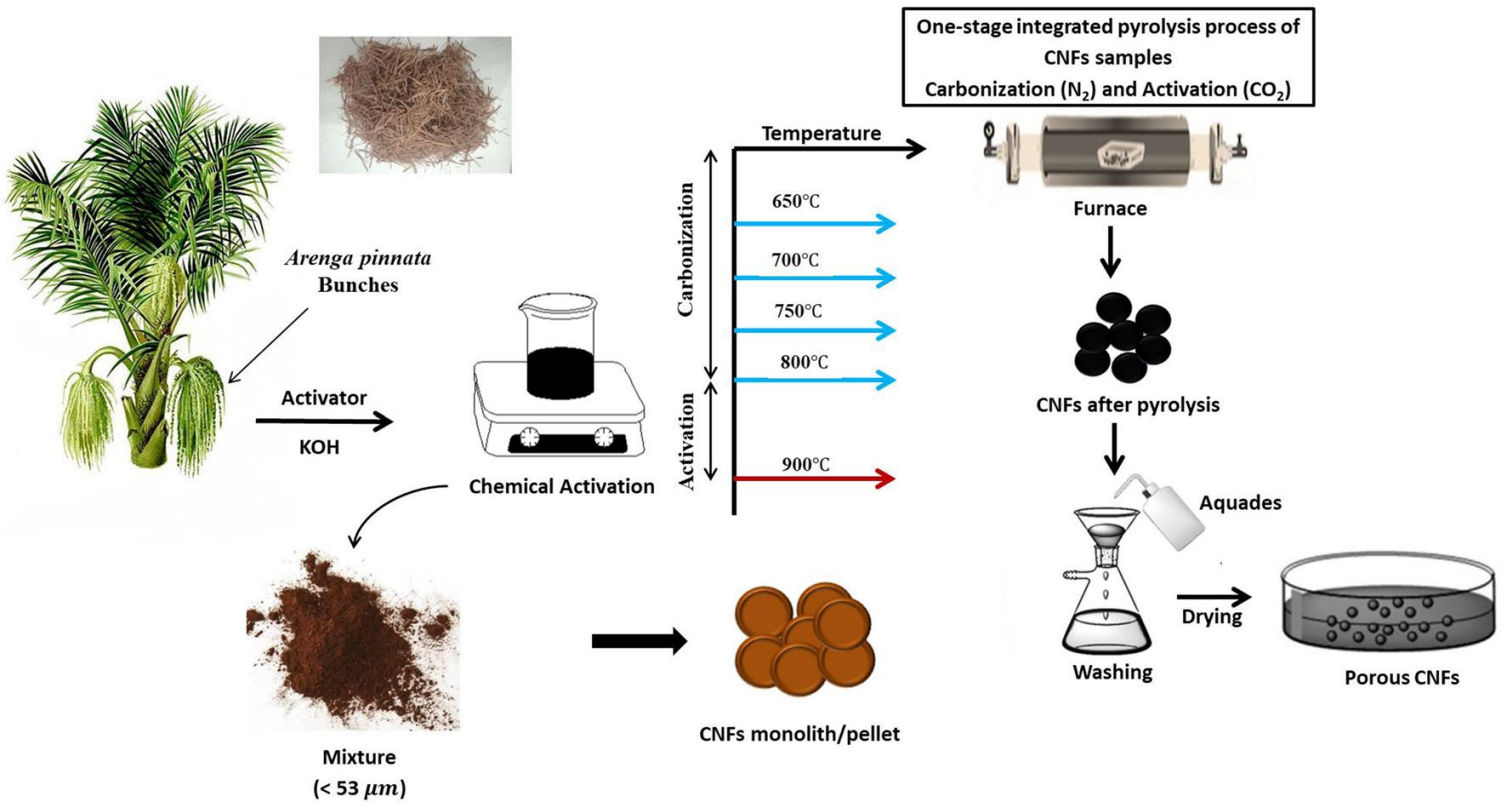
Schematic illustration of the synthesis of carbon electrodes of *Arenga pinnata* bunches.

The *Arenga pinnata* bunches were cut into pieces with a length of ± 4 cm and a diameter of ± 0.1 cm, then cleaned and sun-dried until a constant mass was obtained. Subsequently, the biomass was subjected to pre-carbonization for 1 h at 200 °C, using 0.5 M KOH for chemical activation. The carbon powder was converted into a monolith or pellet form, using a hydraulic press with a pressure of 7 tons. It was followed by carbonization and physical activation and carried out simultaneously in the furnace. The carbonization was performed at temperatures of 650 °C, 700 °C, 750 °C, and 800 °C, with nitrogen gas flowing at 1.5 L min^−1^, for 1 h, while the physical activation process was carried out at 900 °C, with carbon dioxide gas flowing at 1 Lmin^-1^ for 2.5 h. To obtain micro-mesopore distribution, nanofiber structures and wide surface area helped to improve the performance of the supercapacitor cells. The carbon electrodes were then neutralized using aquadest until the pH was neutral (pH ~ 7), and oven-dried at 110 °C, for 24 h. Figure 1 shows the scheme for the synthesis of carbon nanofiber as free-standing electrodes, using *Arenga pinnata* bunches.

## Experimental statement

All the experimental procedures used in this study comply with the relevant institutional, national, and international guidelines and legislation.

### Characterization of physical properties

The physical properties of the carbon electrode were characterized using X-ray diffraction, scanning electron microscopy-energy dispersive X-ray, and Brunauer Emmett tellers analyses. X-ray diffraction characterization was performed to determine the crystalline properties of the carbon electrodes using an XRD Shimadzu 700 instrument with a scattering angle of 15°-60°, a Cu k-α beam source, and a wavelength of 1.5418 Å. Meanwhile, the surface morphology of the carbon electrodes was determined using scanning electron microscopy with an SEM instrument (JEOL JSM-6510 LA), while the constituents of the elements were determined using energy dispersive spectroscopy with an EDX instrument (JEOL JSM-6510 LA). In addition, the surface area and pore size distribution were determined through the adsorbs-desorption method of isothermal nitrogen gas (N_2_) at a temperature of 77 K using quadraSorb station 1 7.01.

### Characterization of electrochemical properties

Several supercapacitor cell components, including stainless steel current collectors, chicken eggshell membrane separators, 0.2–0.3 mm thick carbon electrodes with a diameter of 7–8 mm, were produced using *Arenga pinnata* bunches, and 1 M H_2_SO_4_ as electrolyte. Furthermore, the manufacture of supercapacitor cells and electrochemical characterization were carried out consisting of cyclic voltammetry and galvanostatic charge–discharge methods to determine the specific capacitance. Cyclic voltammetry was carried out with a CV UR Rad-Far 5841 at potentials of 0 to 1000 mV and scan rate of 1 mV s^−1^, 2 mV s^−1^, 5 mV s^−1^, and 10 mV s^−1^. Meanwhile, a galvanostatic charge–discharge analysis was performed using GCD UR Rad-Far 5841 in the potential range of 0–1000 mV and at a 1 A g^−1^ current density.

## Results and discussion

### Microstructure analysis

**Figure 2:**
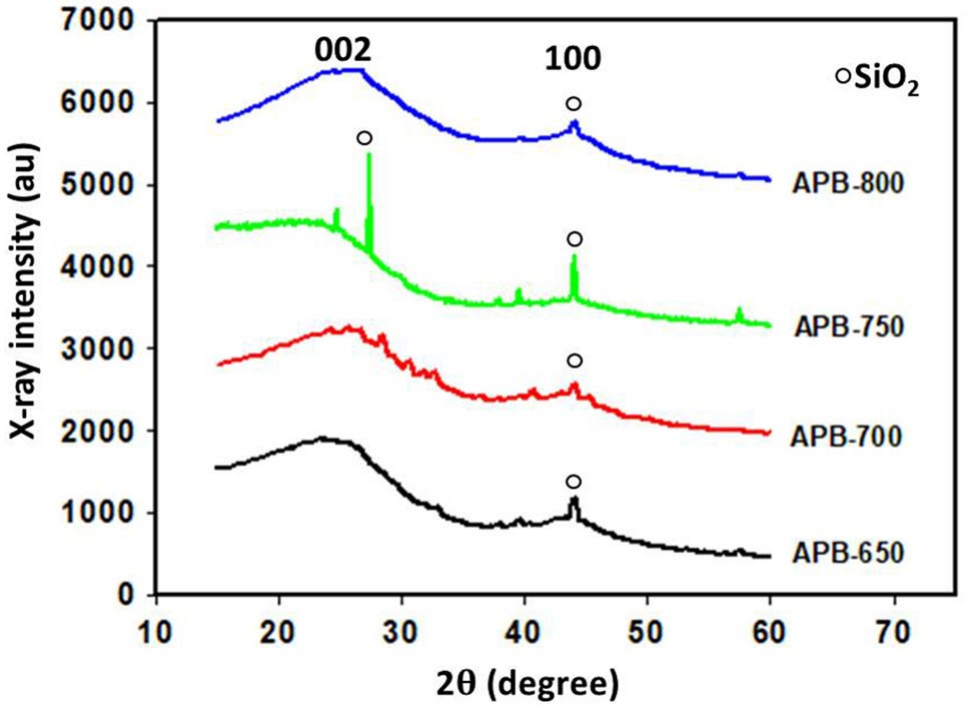
X-ray diffraction pattern of *Arenga pinnata* bunches.

Figure 2 shows the X-ray diffractogram pattern for all APB carbon electrodes based on variations in carbonization temperature of 650 °C, 700 °C, 750 °C, and 800 °C. Based on the diagram, there are two similar diffraction patterns with two broad peaks that are typically carbon materials, appearing at diffraction angles of 24° and 44°, which correspond to the field orientation (002) as well as (100), and depicting irregular or amorphous carbon structures^21^. Furthermore, the APB-650 and APB-750 carbon electrodes in Fig. 2 have sharp peaks, indicating the presence of silicic acid compounds (SiO_2_) found in carbon electrodes. Based on National Bureau of Standards (NBS) data, these elements are detected at angles of 28° and 44°. Silicic acid which possesses crystalline characteristics appeared on the carbon electrode because this compound is a constituent of the *Arenga pinnta* bunch biomass and does not completely decompose during chemical activation, carbonization, and physical activation. The APB-700 and APB-800 carbon electrodes also have a low peak, indicating the presence of trace silicic acid compounds (SiO_2_), which can be ignored^22^.

Table 1 shows the lattice parameters and microcrystalline dimensions for the carbon electrode APB. According to Table 1, the rise in carbonization temperatures led to changes in the L_c_ values and, consequently, the microcrystallite structures. An increase in the carbonization temperature affects the atoms of the carbon electrode, which have a high irregularity with better amorphous characteristics, and this tends to reduce the stack of height (L_c_)^23^. The stack height (L_c_) on the APB carbon electrode is related to its specific surface area based on the empirical formula, namely, SSA = 2/ρ_xrd_L_c_, where xrd = [d_002_ (graphite)/d_002_(sample)] ρ (graphite) with d_002_ (graphite) and ρ (graphite) is 0.33354 nm and 2.268 g cm^−3^^23^. Based on the empirical formula, the L_c_ value and surface area are inversely proportional. If the L_c_ value is high and the surface area is small, otherwise the L_c_ value is low and the surface area is large. APB-700 has the lowest L_c_ value of 9.96 nm, which indicates that the APB-700 samples have the largest surface area compared to the other electrodes.

**Table 1:** Lattice parameters and microcrystalline dimensions of carbon electrodes.

Code samples	2θ		Interlayer spacing		Microcrystalline dimensions		L_c_/L_a_	N_p_
	(002)	(100)	d_002_ (nm)	d_100_ (nm)	L_a_ (nm)	L_c_ (nm)		
APB-650	22.02	42.04	4.03	2.14	51.07	11.23	0.219	2.78
APB-700	25.66	44.45	3.46	2.05	42.11	9.96	0.236	2.87
APB-750	24.83	43.98	3.58	2.03	44.46	10.08	0.226	2.81
APB-800	24.55	43.11	3.62	2.09	47.52	10.17	0.213	2.80

### Surface morphology analysis

**Figure 3:**
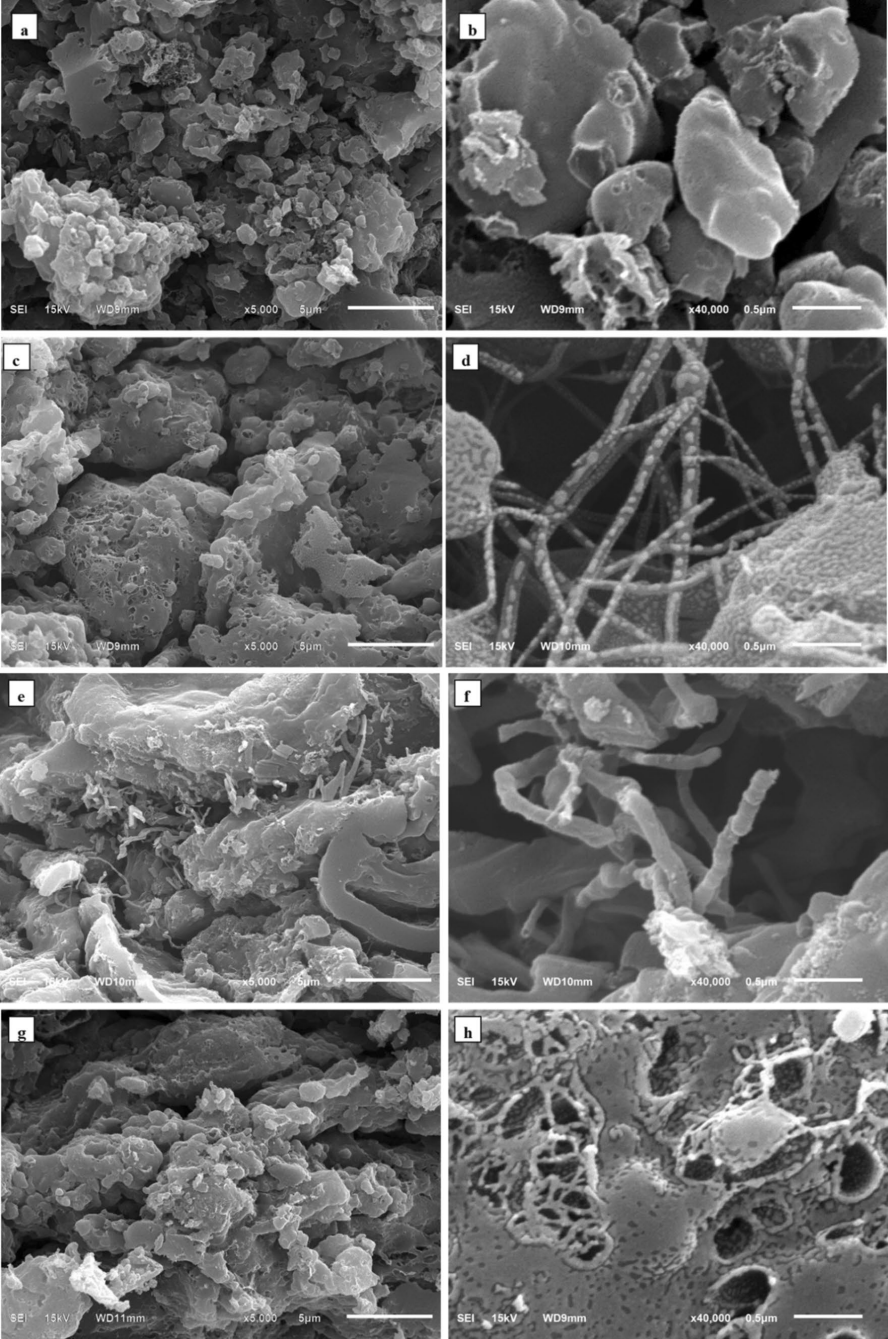
SEM images of **(a)** APB-650, **(b)** enlargement of APB-650, **(c)** APB-700, **(d)** enlargement of APB-700, **(e)** APB-750, **(f)** enlargement of APB-750, **(g)** APB-800, and **(h)** enlargement of APB-800.

Figure 3 show the SEM images of APB carbon electrodes produced at different carbonization temperatures. The electrodes have a relatively developed porous structure with irregular constructs and various sizes, ranging from ten to hundreds of nanometers. APB carbon electrode morphological structures were reviewed based on nutrition and metabolic systems in *Arenga pinnata* bunches, pore channels formed by evaporation of water, and molecular gases produced by thermal decomposition of APB during carbonization^24,25^.

According to Fig. 3a,b, APB-650 has several mesopores with average diameters of 38 nm, as well as several nanofiber structures with an average diameter of 28 nm. Figure 3c,d show the surface morphology of the APB-700 electrode having abundant pores consisting of mesopores with an average diameter of 53 nm, a more regular pore structure, as well as numerous nanofibers with an average diameter of 62 nm. Meanwhile, Fig. 3e,f show the carbon electrodes’ structure collapses in the presence of meso-macropores with average diameters of 81 nm and 126 nm. The nanofibers with average diameters of 134 nm and fiber networks begin to break because the inner wall channels become thinner and adjacent pores interconnect at temperatures above 700 °C^26^. Furthermore, Fig. 3g,h show that the high carbonization temperature of APB-800 causes the fibers to further break down. Thus, only macropores are formed with an average diameter of 224 nm. APB-700 and other electrodes are composed of large amounts of carbon nanofibers that tend to have connections between fibers, causing a rise in specific capacitance. This is because the interfiber connections tend to shorten the charging routes of carbon electrode networks, increasing the charge transfer efficiency and reducing the internal resistance of electrodes^14^.

### The chemical composition analysis

**Figure 4:**
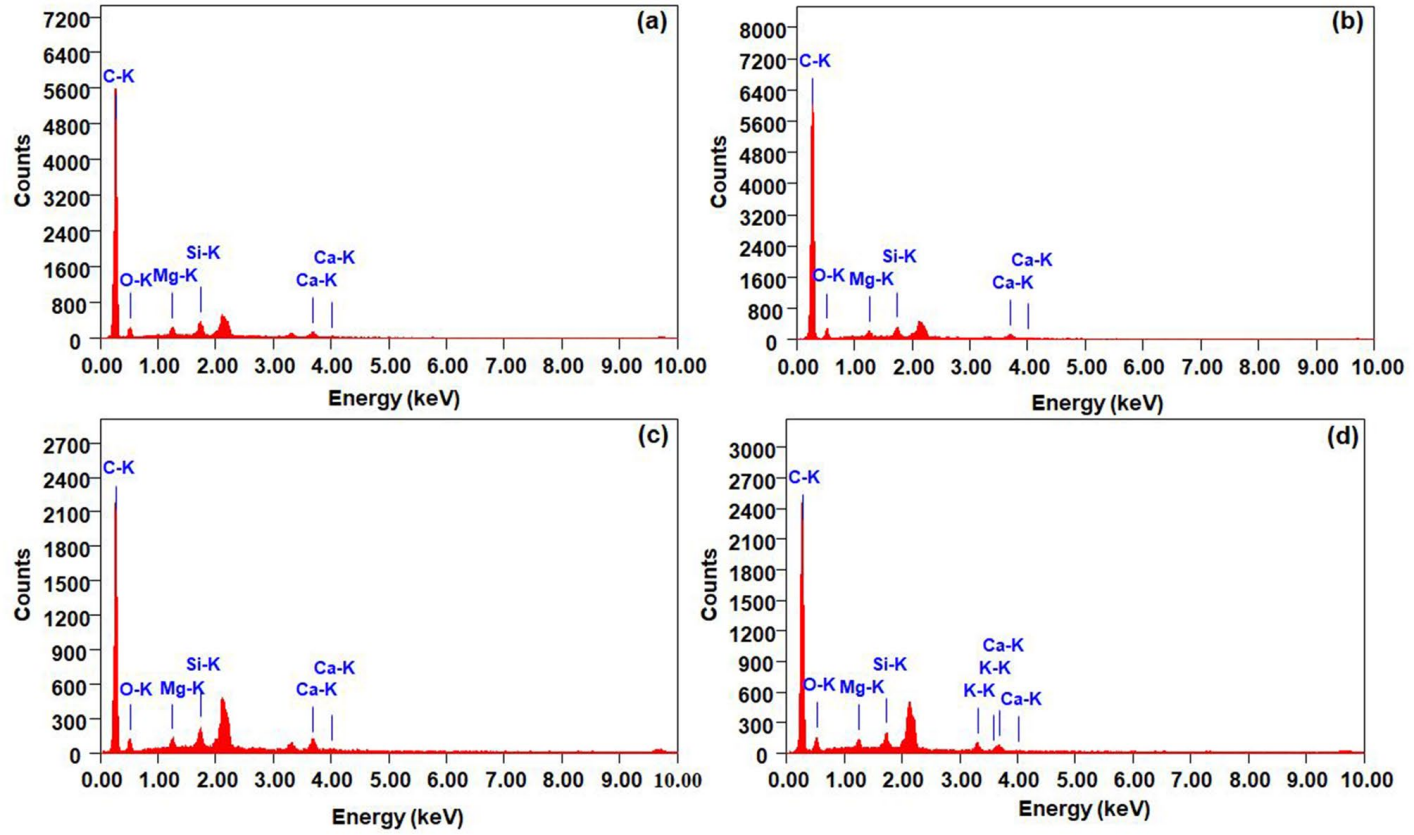
EDX images of **(a)** APB-650, **(b)** APB-700, **(c)** APB-750, **(d)** APB-800.

EDX characterization was used to determine the constituent chemical elements of the APB carbon electrodes. Table 2 shows the elements of the APB-650, APB-700, APB-750, and APB-800 samples. Figure 4 shows carbon is the most prominent chemical constituent depicted by the highest peak. Oxygen is a major constituent element of *Arenga pinnata* bunch biomass, which contains lignocellulosic compounds, including cellulose (C_6_H1_0_O_5_), hemicellulose (C_6_H_12_O_6_), and lignin (C_11_H_14_O_4_). Generally, the APB carbon electrode comprises 9.20% oxygen, however, this value increases in cases where KOH is used in the chemical activation process and CO_2_ is used in the physical activation process. In addition, the potassium compounds originated from the use of KOH in chemical activation, while magnesium, silica, and calcium compounds were from the nutrients contained in the *Arenga pinnata* bunch biomass. Calcium exists because the cell wall of *Arenga pinnata* bunch biomass strengthens the tissue and maintains the integrity of the membrane, while magnesium originates because of its use in sugar and starch synthesis by plants. Furthermore, silica is a constituent of the ash residue of *Arenga pinnata* bunches. Figure 4 shows the sharpest peaks possessed by carbon elements. Compound carbon is the most prominent chemical constituent. APB-700 was discovered to have the highest percentage carbon constituent of 91.72%. Thus, the carbon atoms bond with each other to form double or triple carbon functional groups. The bond is a high conductor that can improve the performance of supercapacitors^27^.

**Table 2:** Elemental of the samples.

Elements (%)	Samples							
	APB-650		APB-700		APB-750		APB-800	
	Weight (%)	Atom (%)	Weight (%)	Atom (%)	Weight (%)	Atom (%)	Weight (%)	Atom (%)
Carbon	86.72	90.88	88.01	91.72	83.60	89.01	83.66	88.91
Oxygen	9.67	7.61	9.00	7.04	11.08	8.86	11.53	9.20
Magnesium	0.88	0.46	0.73	0.38	0.94	0.49	0.90	0.47
Silica	1.54	0.69	1.13	0.50	1.80	0.82	1.15	0.52
Potassium	–	–	–	–	–	–	1.51	0.49
Calcium	1.19	0.37	1.13	0.35	2.58	0.82	1.26	0.40
Total	100%

### Porosity properties analysis

**Figure 5:**
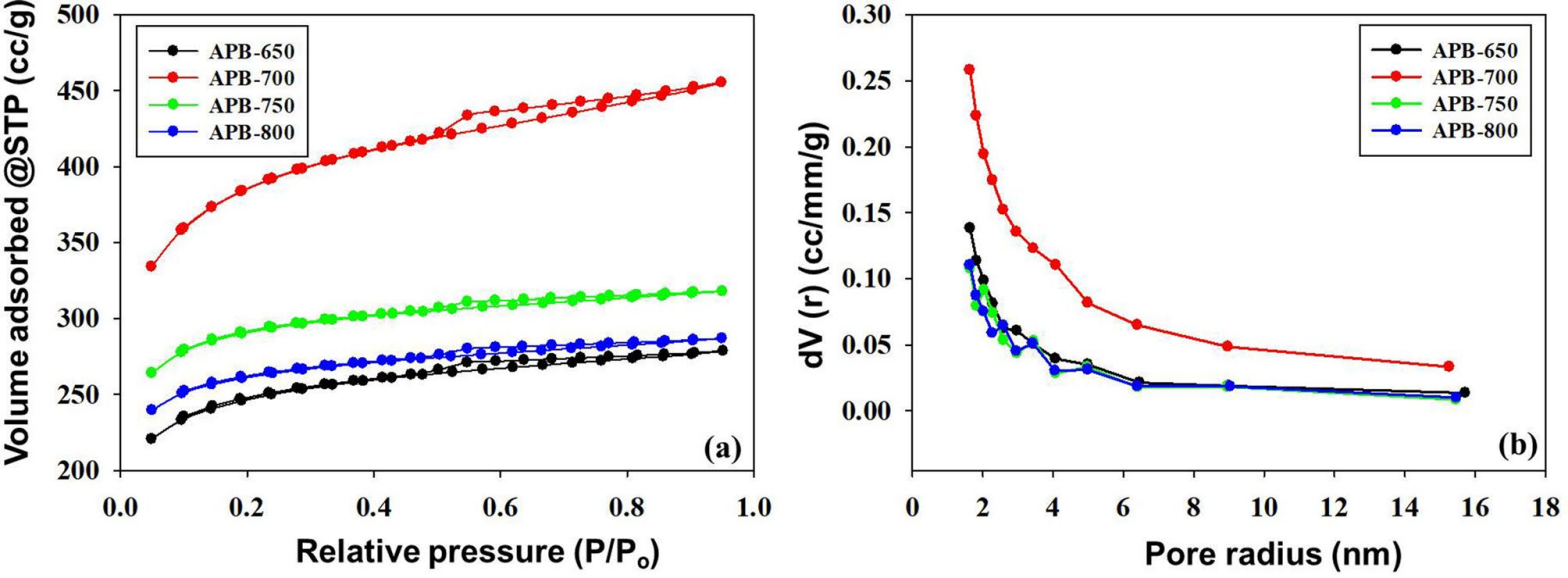
**(a)** N_2_ gas adsorption/desorption of *Arenga pinnata* bunches electrodes and **(b)** pore size distribution of *Arenga pinnata* bunches electrodes.

Figure 5a shows the APB carbon electrode porosity, determined using the Nitrogen isothermal adsorb-desorption method, at a temperature of 77 K. All the carbon electrodes exhibited type I and IV adsorption profiles based on IUPAC classification, with type H1 hysteric loops at P/P0 < 0.1 and H4 at P/P0 > 0.4. These materials have a pore distribution of mesopores and micropore combinations^28^. The hysterical loops vary according to carbonization temperatures 650 °C, 700 °C, 750 °C, and 800 °C, indicating that carbonization temperatures have a significant influence on the development of electrode porosity during physical activation.

**Figure 6:**
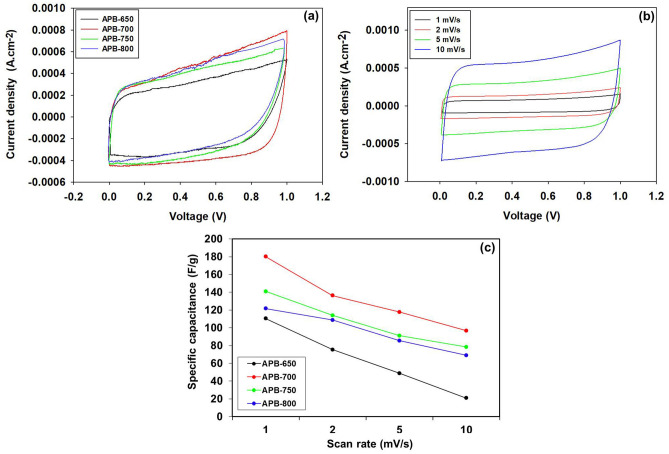
**(a)** Cyclic voltammetry curve of sample APB, **(b)** cyclic voltammetry curve of APB-700 at different scan rate, **(c)** specific capacitance vs scan rate of sample APB.

Meanwhile, the isotherm curves at 750 °C and 800 °C indicate imperfect H4-type hysterical curves, characterized by mesopores formed like ink bottles and narrow neck pores, involving complex mechanisms including pore blockage, dislocation, or cavitation. The capillary condensation of pore tissue is blocked by the narrowing of pores, resulting in a lower desorption rate. Furthermore, steam pressure desorption depends on the pore size, surrounding pore state, and tissue configuration^29^. Thus, a carbonization temperature of 700 °C causes the pores to shrink and form a whole mesopore, indicating an ideal type IV hysterical loop.

Figure 5b shows the pore size distribution of APB carbon electrodes. All APB carbon electrodes have a combined micropore (< 2 nm) and mesopore (2–10 nm) pore distribution. The APB700 samples showed pore dominance, with pore sizes below 4 nm, followed by APB 750 and APB 800. Pore distribution affects the transfer of electrolyte ions on the surface of the electrode. The macropores serve as channels for easy access to electrolytes; however, these pores are not primarily responsible for the increase in specific capacitance. Meanwhile, mesopores act as a medium for electrolyte ion diffusion, and microporesstore electrolyte ions to increase the specific capacitance. Therefore, any increase in capacitance is significantly due to an increase in the meso- and micropores of carbon electrodes^14^.

In addition, the APB carbon electrode surface areas were 779.118 m^2^ g^−1^, 1231.896 m^2^ g^−1^, 909.558 m^2^ g^−1^ and 814.891 m^2^ g^−1^ for carbonization temperatures of 650 °C, 700 °C, 750 °C, and 800 °C, respectively. This shows that a moderate rise in temperature successfully increased the specific surface area and the total pore volume of carbon electrodes^29^. However, higher carbonization temperatures easily increase the area of micropores. APB-700 was discovered to have the optimum S_BET_ value of all the carbon produced, while APB-750 and APB-800 electrodes show lower values, as increased carbonization temperatures tend to damage pore walls further, causing widened pore size and collapse of micropore pore structure, resulting in decreased surface area.

### Cyclic voltammetry analysis

Cyclic voltammetry analysis was carried out to determine the specific capacitance of supercapacitor cells using a scan rate of 1 mVs^-1^ with a voltage of 0–1 V. Figure 6a shows a cyclic voltammogram curve with the same potential window. A repeating cycle with a curved shape approaching a rectangular shape was formed and indicated ideal EDLC properties for each electrode^30^. Supercapacitor cells have similar reversible, pure electrostatic, and nonpseudocapacitance reaction mechanisms, as well as stability in acidic solutions^31^. The potential window has a similar shape, however, the curve area of each sample is different due to the variation in carbonization temperatures. The area formed on the curve within the same scan rate is affected by the charged and discharged cycles. In the supercapacitor, the electrochemical cell produced a large charged-discharged current density. Therefore, a wider curve was obtained, indicating a high specific capacitance value. The relationship between the current density value and the specific capacitance is given by the empirical formula C_sp_ = I/(s.m)^32^.

Table 3 shows the specific capacitances of the APB carbon electrodes. APB-700 was discovered to possess the optimum specific capacitance of supercapacitor cells. A higher carbonization temperature will accelerate the reaction between carbon, oxygen, and hydrogen, causing the release of nitrogen, which is bonded to oxygen and hydrogen regardless of carbon (dissociation reaction). The dissociation reaction causes a decrease in charcoal oxygen and hydrogen, resulting in mass shrinkage of the carbon samples. The shrinkage results in a reduced density and multiplies the pore wells. Thus, numerous pores are formed. There is a higher capacity to accommodate anions and cations. The increased number of ions is trapped within pore wells, which increases the specific capacitance of supercapacitor cells. The APB-750 and APB-800 supercapacitor cells show low specific capacitance compared to APB-700 due to higher carbonization temperatures. This causes damage to carbon electrodes, including collapsed carbon electrode structures, resulting in thinner inner wall channels and interconnecting pores caused by excessive dissociation reactions.

**Table 3:** Specific capacitance values using the CV method at a scan rate of 1 mV s^−1^.

Sample code	Ic (A)	Id (A)	Mass (gr)	C_sp_ (F g^**−**1^)
APB-650	0.00046	−0.00026	0.006	111
APB-700	0.00066	−0.00042	0.006	181
APB-750	0.00054	−0.00031	0.006	142
APB-800	0.00050	−0.00029	0.006	132

**Figure 7:**
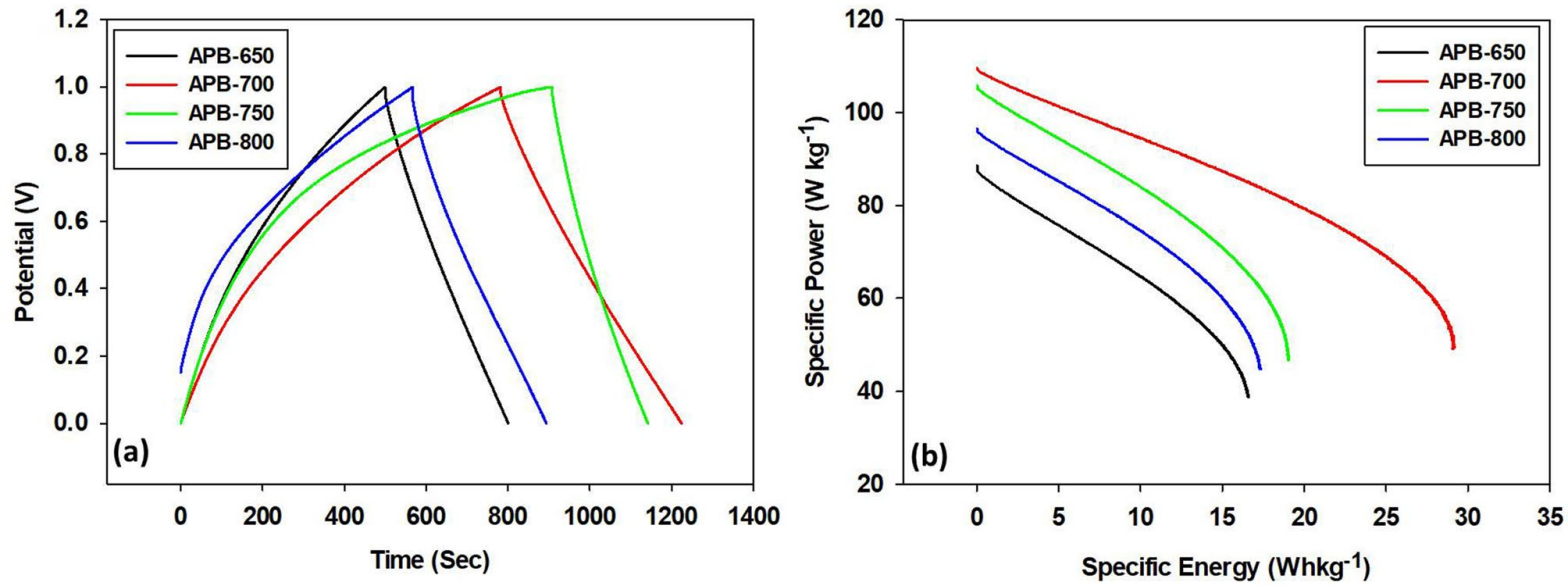
**(a)** GCD curves of APB, **(b)** Ragone plots for APB-650, APB-700, APB-750, and APB-800.

Voltammetry cyclic instruments in APB-650, APB-700, APB-750, and APB-800 supercapacitor cells were treated with different scan rate of 1 mVs^-1^, 2 mVs^-1^, 5 mVs^-1^, and 10 mVs^-1^ to determine the effect of the scan rate on the capacitance value obtained. In addition, the scan rate dramatically affects the shape of the curve and the specific capacitance. Figure 6b shows that the specific capacitance value decreases with the increasing scan rate. The voltage administration rate becomes slower, impacts the dissociated electrolyte ions to diffuse into the pores of carbon electrodes, and forms a double layer^33,34^. Similarly, high scan rate reduce the time required for dissociated electrolyte ions to diffuse into carbon electrode pores. A voltage of 0–1 V (0–1000 mV) at a scan rate of 1 mV s^−1^ requires 1000 s to completely diffuse the ions into the electrodes. While the scan rate of 10 mV s^−1^ requires 100 s. Therefore, the difference in diffusion time is based on the scan rate, and a long diffusion time increases the specific capacitance. The curve maintains a rectangular shape and shows a slope, especially at a scan rate of 10 mVs^-1^ due to an increase in ohmic and kinetic diffusion ions^35^. Figure 6c shows the trend of the scan rate on the specific capacitance.

### Galvanostatic charge–discharge analysis

Figure 7a shows a comparison of the GCD curves for supercapacitor cells APB-650, APB-700, APB-750, and APB-800 in the potential range of 0–1 V and at a 1 A g^−1^ current density. All samples have a triangular shape curve, indicating ideal EDLC properties for each cell, high efficiency, and good reversibility in the charge–discharge process. However, each sample’s charge–discharge time differs, with values of 8.007 s, 12.246 s, 11.421 s, and 8.939 s for supercapacitor cells of APB-650, APB-700, APB-750, and APB-800, respectively. The charging and discharging times affect the specific capacitance value. Therefore, longer charge and discharge times indicate that a higher number of electrons and electrolyte ions participate in these processes on the electrode surfaces. Meanwhile, an increase in the number of electrons and electrolyte ions trapped within these electrode surface pore wells increases the supercapacitor cell-specific capacitance.

The specific capacitances of the APB-650, APB-700, APB-750, and APB-800 supercapacitor cells are 117.5 F g^−1^, 201.6 F g^−1^, 171.6 F g^−1^, and 132.5 F g^−1^, respectively. This corresponds to the values obtained using the cyclic voltammetric method, where APB-700 and APB-650 were discovered to possess the highest and lowest specific capacitance values, respectively. Furthermore, a rise in carbonization temperature is able to excessively dissociate the existing pore structure and is, therefore, able to damage the previously formed pore arrangement and erode pore walls, thus increasing the poor size and decreasing the sample’s electrochemical properties^36^.

The carbonization temperature (700 °C) was discovered to have the optimum effect on the APB-700 sample, where an increase in the number of pores distributed at the electrode surface occurred, thus removing any barrier to the ion accessibility at the electrode–electrolyte interface and producing relatively higher supercapacitive properties compared to other samples. This is also supported by the SEM characterization results, indicating that the APB-700 sample has the largest specific capacitance data, where the pore structure of APB-700, composed of large carbon nanofiber contents, tends to have connections between fibers because interfiber connections tend to shorten the carbon electrode network’s loading routes, increase charge transfer efficiency, reduce the electrodes’ internal resistance, and consequently cause a rise in specific capacitance. In addition, the BET data also confirm that the APB-700 sample has the largest surface area, where the specific capacitance value is directly proportional to the surface area. Table 4 shows a comparison of each variation in carbonization temperature of different biomass materials concerning specific capacitance.

**Table 4:** Comparison of variation in carbonization temperature of different biomass materials.

Biomass materials	Carbonization temperature ($^\circ{\rm C}$)	Optimum carbonization temperature ($^\circ{\rm C}$)	C_sp_ (F g^−1^)	References
*Laminaria japonica*	600, 800, 1000, and 1200	800	192	^2,6^
Bamboo waste	600, 700, 800, and 900	800	174	^3,8^
*Syzygium oleana *leaves	500, 600, and 700	700	188	^30^
Oil palm empty fruit bunches	400, 500, 600, and 700	500	112	^3,2^
Oil palm empty fruit bunches	600, 700, and 800	800	80	^3,9^
*Arenga pinnata* bunches	650, 700, 750, and 800	700	201.6	This work

Figure 7b shows the Ragone plot between the specific power variations and the specific supercapacitor energy of samples APB-650, APB-700, APB-750, and APB-800. These values decrease linearly with specific energy searches for all APB cells, implying that less energy is released at higher power outputs. APB-700 cells possess a better energy and power relationship than other APB cells because a carbonization temperature of 700 °C is able to result in better electrode performance for supercapacitor cell applications. Furthermore, the specific energy and maximum specific power for the APB-700 sample are 28.0 Wh kg^−1^ and 109.5 W kg^−1^, respectively. These results are comparable to the values reported for supercapacitor-type electrodes derived from other biomass precursors, such as syzygium oleana leaves, with values of 26.0 Wh kg^−1^ and 96 W kg^−1^, respectively, at the optimum carbonization temperature of 700 °C^29^.

## Conclusion

In this study, porous carbon nanofibers were successfully prepared using *Arenga pinnata* bunches as raw material. This is the first study where pure biomass alone was used as the main material for making carbon free-standing electrodes without employing the heteroatom doping, binder, electrospinning, template method, and composites, or other synthetic materials. Furthermore, APB-700 was reported to possess the highest specific surface area and specific capacitance of 1231.896 m^2^ g^−1^ and 201.6 F g^−1^, respectively, with the combination of the mesopore and micropore nanofiber morphological structures for the two electrode systems. Therefore, *Arenga pinnata* bunches are suitable sources of carbon-rich biomass for the synthesis of porous carbon nanofiber materials as high-performance supercapacitors as free-standing electrodes and energy storage.
